# The Wave–Particle Dualism of Photons as Seen from an Informational Point of View

**DOI:** 10.3390/e27101037

**Published:** 2025-10-03

**Authors:** J. Gerhard Müller

**Affiliations:** Department of Applied Sciences and Mechatronics, Munich University of Applied Sciences, D-80335 Munich, Germany; gerhard.mueller@hm.edu

**Keywords:** quantum measurement, energy dissipation, information gain, elementary observations, observer participance, continuum idealization, existence, reality

## Abstract

This paper deals with J. A. Wheeler’s proposal that each piece of reality owes its existence to observation—an approach to physics, which implies that all physical entities at their bottom are informational in character. Focusing on the double-slit experiment with photons, which is the key evidence for the wave–particle dualism of photons, this paper follows Wheeler’s observational approach and interprets this experiment as a question posed to nature. Considering how the enquiry regarding the wave–particle duality of photons is answered by nature, it is shown that experimental questions are being answered by nature in the form of spatiotemporal patterns of elementary observations (EOs) which are binary pieces of information, produced by the dissipation of energy. Working through this line of thought, Wheeler’s statements of “binary information gain”, “observer participance” and the “impossibility of continuum idealizations of physical laws” are elucidated and connections to the Landauer Principle are made.

## 1. Light: Waves or Particles?

**Figure 1:**
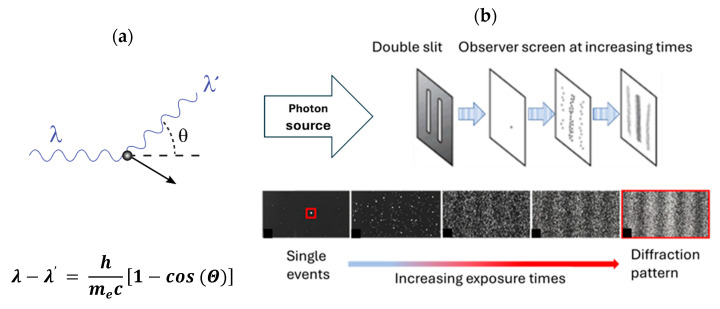
(**a**): An X-ray photon of wavelength λ comes in from the left, collides with an electron at rest, and a new photon of wavelength λ′ emerges at an angle θ. The electron recoils, carrying away an angle-dependent amount of the incident photon momentum [9]. (**b**): (**top row**) Sketch of a double-slit experiment with photons (DSE), conducted for increasingly longer periods of time. Photon impacts on the detector screen feature as black dots which turn into increasingly denser patterns of black dots as time proceeds. After development of the photographic plates, the individual “photon impacts” appear as small, permanently whitened spots, approximating “diffraction patterns” in the long run [10].

Throughout the history of science, the nature of light was an intensely debated subject that kept famous scientists busy over several centuries up to the present time. In the 17th century, i.e., upon the onset of modern science, two opposing views concerning the nature of light existed: while Isaac Newton [1,2] advocated a corpuscular nature of light, Christiaan Huygens [3] favored a wave-like picture of light. This controversy turned towards an overwhelmingly accepted wave nature of visible light when Thomas Young published the results of his interference experiments in 1807 [4]. Another piece of evidence in favor of the wave nature of light was the observation of Poisson spots [5,6], i.e., bright spots of light in the center of dark shadows of circular obstacles blocking off visible light sources. This general belief in the wave nature of light was shattered in the early 20th century when Max Planck [7] showed that the frequency spectra of the thermal radiation emitted from black bodies could only be satisfactorily explained on the assumption that light is exchanged between the cavities and the heated walls of black-body sources in the form of light quanta of sizes $E_{ph}=h\nu$, where $E_{ph}$ stands for the energy of the light quanta, ν for the frequency of light and $h=1.034\times 10^{-15}\;eVs$ for the elementary quantum of physical action. In his Nobel Prize paper of 1905 [8], Einstein generalized the idea of light quanta to all those occasions in which light was generated, absorbed or converted into different forms, such as in luminescence interactions or in the photo-electric effect. With freely propagating light still proceeding in a wave-like manner, Einstein proposed that light behaves much more like a corpuscular particle when it is generated, absorbed or turned into different forms. Einstein’s paper, therefore, can be viewed as the first statement of the wave–particle dualism of light. Key experiments that later confirmed the dual nature of light were the Compton scattering experiments of X-rays on electrons [9] and interference experiments with photons passing in a one-by-one manner through a two-slit diaphragm on their way towards a photographic detection screen [10]. For the sake of clarity and further discussion, both experiments are sketched in Figure 1a,b below.

While the Compton scattering experiments showed that X-ray photons can transfer momentum to an electron much as if two corpuscular pieces of matter had collided with each other [9], the double-slit experiment with photons (DSE) [10] indicated that traveling photons can freely alternate between particle- and wave-like motion within one single experiment. Following Einstein’s arguments [8], photons are emitted from the source in Figure 1b in corpuscular form and then proceed through open space in an undulatory manner until they hit the double-slit obstacle, where their motion becomes broken up into several streams of diffracted wave trains which finally interact with the detection screen. Once they have arrived there, the photons are absorbed in a corpuscular form, thereby producing seeming “particle impacts” with a spatial distribution characteristic of a classical diffraction pattern.

While the above narrative of a wave–particle duality is largely rooted in the era of “old quantum theory”, it is surprising to see that after more than 20 years of “old quantum theories”, two radically different versions of “modern quantum theories” emerged almost simultaneously: Heisenberg’s matrix mechanics [11,12,13] and Schrödinger’s wave theory [14,15,16,17,18]. In view of the huge differences in mathematical appearance of both theories, an intense discussion arose in the years 1925–1927, with an intent of arriving at a physical interpretation of the mathematical content of both theories. This work, particularly driven by Bohr, Heisenberg and Born, led to the so-called “Copenhagen interpretation” of quantum mechanics [19,20], which became a foundational concept in quantum mechanics. Central ideas leading to the Copenhagen interpretation are Born’s statistical interpretation of the Schrödinger wave function [21], Bohr’s complementarity principle [22] and Heisenberg’s uncertainty relationships [23].

Following Bohr’s complementarity principle, quantum objects like electrons and photons feature pairs of complementary properties, such as position and momentum, or complementary forms of existence such as wave-like and particle-like modes of propagation, which cannot simultaneously be observed. While quantum objects normally abound in a super positional state of indeterminacy, their complementary properties only become manifest once they are actually observed in a specifically designed experiment. While in such an interaction the first property in this pair becomes fixed, the second is rendered unobservable and questions regarding its momentary state are turned meaningless. Overall, this concept implies that quantum-mechanical measurements select, out of a cloud of possibilities, one specific outcome through a process which had become generally known as “wave function collapse” [24,25].

Coming from the world of classical mechanics and electrodynamics where sharply localized particles and continuously distributed waves are key theoretical concepts and supposedly primary forms of existence, both the semi-classical and the fully quantum-mechanical interpretations of the DSE lead to the same dilemma of a wave–particle duality of photons that embraces two mutually incompatible forms of existence [26,27].

## 2. Observation Creates Reality

An interesting step forward was taken in 1989 by John Archibald Wheeler [28], who asked the question of “How comes existence?”. In his seminal paper he proposed that every piece of reality owes its existence due to observation, an idea which he epitomized as IT from BIT. Reading through Wheeler’s paper, his view of an observation-based physics is no less mind-boggling than the more familiar concept of a wave–particle duality [26,27] as it deeply touches on the assumption of a pre-existing material world that exists independent of human existence. In order to reveal the mental challenges imposed by Wheeler’s approach, we simply quote the four main conclusions in his seminal paper [28]:

*“(1) The world cannot be a giant machine, ruled by any preestablished continuum physical law; (2) There is no such thing at the microscopic level as space or time or spacetime; (3) The familiar probability function … of standard quantum-mechanical theory provides a mere continuum idealization … that conceals the information-theoretic source from which it arrives; (4) No element in the description of physics shows itself closer to primordial than the elementary quantum phenomenon, i.e., the elementary device-intermediated act of posing a yes-no physical question and eliciting an answer or, in brief, the elementary act of observer participance. Otherwise stated, every physical quantity, every “IT”, derives its ultimate significance from “BITs”, i.e., binary yes-no indications, a conclusion which we epitomize in the phrase “IT from BIT”.*

Wheeler’s “IT from BIT” statement opened a controversial discussion confronting the innovative “IT from BIT” vs. the more classical “BIT form IT” approaches [29,30,31,32,33,34]. In the following sections, we follow Wheeler’s general reasoning and apply his ideas to the well-researched and widely known example of light quanta passing through a double-slit diaphragm and detected by photographic means after their passage through the double-slit obstacle. By considering this specific example, we attempt to clarify Wheeler’s notions of “binary information gain”, “observer participance” and “continuum idealizations of physical laws”.

## 3. Experimentally Posing Questions and Receiving Answers

Concerning the nature of light and photons, the key question is “How do photons propagate through space and how is their propagational behavior influenced by obstacles on their way towards a detection screen where their arrival is indicated?”

In designing an experiment that could potentially answer this question, the basis is resorting to one of those ideas about light and photons that had emerged over time as described in Section 1. Choosing as a basis Bohr’s theory of complementarity, photons as quantum objects normally abound in a state of super-positional indeterminacy, failing to observably display any of those properties out of a pair of complementary properties.

The DSE deals with this situation by setting up an experiment in which single photons are sequentially emitted from the source in a state of indeterminacy and injecting them into a parcourse of obstacles which challenge the photons with regard to displaying either of their two complementary properties. In the DSE the first obstacle is the double-slit diaphragm itself. Upon traversal through this obstacle, each photon is challenged towards exhibiting its wave nature. This first question is answered by splitting the incoming photon wave function into several streams of diffracted wave trains which travel onwards towards the downstream detection screen. Upon arrival there, each wave train becomes challenged with regard to converting with some finite probability to its complementary particle form. This latter challenge is answered by producing an apparent “particle impact” on the detection screen. By processing increasing numbers of photons through the DSE, more and more apparent “particle impacts” emerge at seemingly random positions on the detection screen, until the spatial patterns of apparent “particle impacts” finally resemble those “classical diffraction patterns” that had proposedly been formed upon traversing the double-slit obstacle.

These latter forms of “granular diffraction patterns” (GDP) on the photographic detection screen represent the final experimental answers that can be provided by a DSE. Overall, this particular example indicates that complex experimental answers discontinuously and sequentially build up from more elementary answers, a situation which is strongly reminiscent of technical communication scenarii in which meaningful texts and images are transmitted through a technical information channel in the form of meaningless binary digits or bits.

## 4. Experimental Answers Are Digital

In this section we return to Wheeler’s proposal that “*all physical entities, at their deep bottom, are informational in character*”.

Focusing on the first photograph on the left-hand side in the bottom row of Figure 1b, only one single white spot in a black background can be observed. As this observation can neither be unequivocally attributed to a “particle impact”, nor to a “diffraction pattern”, the only unbiassed interpretation, possible at this stage, is that “an event had happened at the spacetime location $\left[\vec{r},t\right]$ at which a photon had interacted with the photographic detection screen”. At this point, it is relevant to note that the observation of such an event is meaningless in the sense that its observation does not yield any information, other than that, that a simple yes–no alternative of the form

(1) $$\left\{\frac{yes}{no}\right\}=\left\{\frac{event\;has\;happened\;at\left[\vec{r},t\right]}{event\;has\;not\;happened\;at\left[\vec{r},t\right]}\right\}$$

had been decided. As deciding a simple alternative, as for instance by tossing a coin, yields the minimum possible amount of information, the observation of a single bright spot may rightly be called an “elementary observation”, an “EO”, or simply a quantum of observation [35,36]. Referring back to Figure 1b, it is evident that the EOs are observables of a macroscopic size which can be observed either visually or at least with the help of an optical microscope, which implies that these EOs occupy areas of sizes $A_{EO}≅{\frac{\pi }{4}d}_{EO}^{2}$, where the effective diameter $d_{EO}$ is larger than the Abbe ‘diffraction limit [37]

(2) $$d_{EO}\;\geq \frac{\lambda }{N_{air}\;sin(\theta )}.$$

In this latter equation, λ is the wavelength of visible light, $N_{air}\sim 1$ the refractive index of air and θ the half angle subtended by the optical objective lens. With this understanding in place, the amount of information gained in observing a single EO amounts to gaining one bit of cognitive information, a unit of information to be carefully distinguished from the measure of thermodynamic information, as earlier established in Leo Szilard’s 1929 paper [38] and later used in the development of a novel theory of statistical thermodynamics based on information [38,39,40].

Processing in a sequential manner $N_{ph}$ photons through the DSE, $N_{ph}$ EOs will emerge in small areas of size $A_{EO}$, centered around positions with Cartesian coordinates $\left(x_{i},y_{j}\right)$ on the photographic detection screen of size $A_{screen}$. In this way, huge response matrices EO will emerge with $N_{EO}=\;A_{screen}/A_{EO}$ elements, where each element ${EO}_{i,j}$ counts the numbers of EOs that had appeared in the vicinity of $\left(x_{i},y_{j}\right)$ and ${EO}_{i,j}=0$ otherwise. With each ${EO}_{i,j}$ representing a piece of information of one cognitive bit, the seeming “particle impacts”, accumulated on the photographs in the bottom row of Figure 1b, are turned into matrices EO, representing 2D granular arrays of cognitive bits. Normalizing the matrix elements with regard to the largest element inside the matrix, a second matrix, PEO, will emerge which enumerates the probability ${PEO}_{i,j}$ that an EO will emerge at a position $\left(x_{i},y_{j}\right)$ when a single photon is processed through the DSE. Finally, concentrating on a particular line in the matrices EO or PEO, a 1D diffraction pattern re-appears that resembles those continuous diffraction patterns that represent the probability distributions of “particle impacts” on the detection screens of DSEs as discussed in many textbooks [10].

## 5. How Does Information Turn Observable?

So far, we have only made a connection between apparent “particle impacts”, on the one hand, and binary pieces of information, on the other hand. In this discussion we have left open the question of how such binary decisions, which are taken on the unobservable small scale of quantum phenomena, actually become observable on a human-observable macro-scale. A complicating factor in the pursuit of this question lies in the complexity of those photochemical processes that take place inside the photographic detection screens. A problem of particular relevance is where in this process chain does the transition actually take place, and where does the microscopic process of “wave function collapse” end and turn over into a chain of classical processes that turn the initiating quantum-mechanical wave function collapse into a macroscopically observable event.

In order to make progress in this direction, we leave the realm of photochemistry and turn to our previous work on photo-ionization detectors (PID) [41]. The reason for making this choice is that, firstly, the physics of PIDs can be easily overseen, and that, secondly, PIDs are able to perform as ideal photon detectors as explained in the textbook of Kingston [42]. PIDs, therefore, form ideal conceptual devices, which reveal how unobservable photon–detector interactions, occurring on the length scale of quantum phenomena, can be turned into macroscopically observable EOs [35,36]. Key motivation for considering such devices is elucidating Wheeler’s statement number 4, namely that

*“No element in the description of physics shows itself closer to primordial than the elementary quantum phenomenon, i.e., the elementary device-intermediated act of posing a yes-no physical question and eliciting an answer or, in brief, the elementary act of observer participance.”*

**Figure 2:**
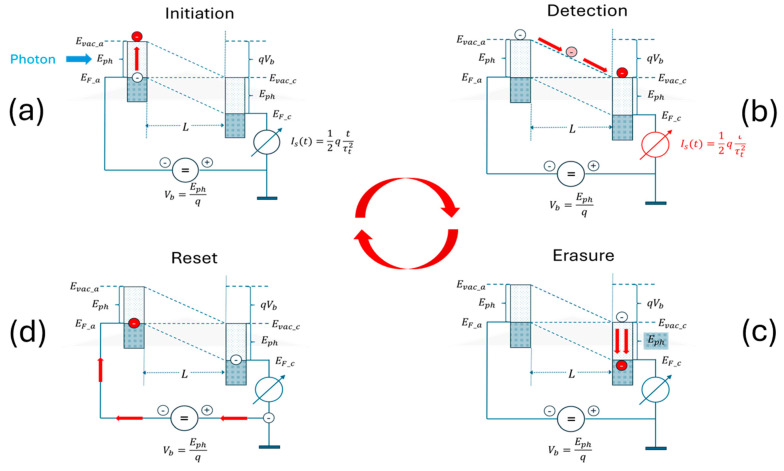
Architecture of a photo-ionization detector (PID) as shown in the form of a semiconductor-like band diagram. The four sub-figures indicate how a photoelectron, liberated from the photocathode on the left (**a**) moves through the device, thereby creating a triangular current pulse $I_{s}(t)$ (**b**). (**c**) shows that upon termination of the current pulse, the energy $E_{ph}$ carried by the incoming photon and the kinetic energy $E_{kin}=qV_{b}=E_{ph}$, gained by the liberated photoelectron on its journey towards the photoanode, are both dissipated inside the photoanode and converted there into low-temperature heat at the detector temperature $T_{D}$. (**d**), finally, shows that the thermalized photoelectron needs to be lifted back to the Fermi energy of the photocathode $E_{F,a}$ to enable a new round of photon detection.

The principle architecture of a PID is shown in Figure 2 in the form of a semiconductor-like band diagram, featuring two metal or semiconductor electrodes positioned face-to-face to each other in the form of a parallel-plate capacitor, with both electrodes having an electron work function of $q\phi_{m}\leq E_{ph}$ which is smaller than the energy $E_{ph}$ of the photons to be detected. The four subfigures in Figure 2 show how a photon, entering from the left, generates a photoelectron that is able to move from photocathode to photoanode through an electrode gap of width L, thus generating a displacement current $I_{s}(t)$ which is macroscopically observable outside the device and which indicates that a photon had arrived at the spacetime location $\left[\vec{r},\;t\right]$ of the PID and interacted there, within a volume of size $\Delta V=L^{3}$ and within a time duration of length $\Delta t=\tau_{t}$, where

(3) $$\tau_{t}=\frac{L}{c}\sqrt{\frac{2m_{e}c^{2}}{qV_{b}}}$$

is the transit time through the electrode gap. In this latter equation, L is the electrode gap width, c the speed of light, $m_{e}c^{2}$ the electron rest mass, q the electronic charge and $V_{b}$ the bias voltage applied across the electrode gap.

Overall, Figure 2 shows how an unobservable photon–matter interaction in which an electron is raised from the Fermi energy of the photocathode to the vacuum level of this same electrode is turned into a macroscopically observable event. The interesting item revealed by Figure 2 is that the initiating photon–photocathode interaction can be regarded as a form of wave function collapse and the classical follow-on processes acting upon the liberated photo electron as amplification processes that make the unobservable initiating event macroscopically observable. These classical follow-on processes, in particular, show that amplification requires observer-related energy resources to be transferred to the conceptional device (Figure 2b) and that the photon energy $E_{ph}$ and the kinetic energy $E_{kin}=qV_{b}\approx E_{ph}$ that had been supplied to the photoelectron both need to be dissipated and dispersed in the environment to avoid a pile-up of heat inside the photoanode. Finally, in case a periodically repeatable operation is to be achieved, the equilibrated photoelectron needs to be removed from the Fermi energy of the photoanode back to the Fermi energy of the photocathode, thereby increasing the energy expense for observation.

With this brief digression into the quantum-mechanical measurement problem, we turn to the problem of measuring the observational value of an EO in terms of physically measurable parameters. As already shown in our previous work [35,36], EOs are pieces of physical action, generated at the expense of creating entropy and representing simple yes–no answers indicating whether a photon had interacted with the PID or not. In particular, we have shown that the EOs, generated by a PID, can be characterized by two dimensionless figures of merit (FOM) with the first FOM signifying the observational value of the generated EO

(4) $${OV}_{EO}=\frac{\frac{1}{3}qV_{b}\tau_{t}}{h}.$$

and the second its statistical significance

(5) $${SI}_{EO}=1-1/SN(E_{ph},L,V_{b},T_{D}),$$

that the detected EO had not been generated by a random thermal fluctuation inside the device itself.

While the first FOM measures the macroscopic observability of the EO in terms of the physical action $W_{obs}=\frac{1}{3}qV_{b}\tau_{t}$ that had been generated in step b and as measured relative to the physical action h that had been generated in step a, the numerical value of the second FOM depends on the magnitude of the signal-to-noise ratio

(6) $$SN\left(E_{ph},T_{D},L,V_{b}\right)=\frac{1}{\surd \pi }\sqrt{\frac{\left[\frac{E_{ph}}{k_{B}T_{D}}\right]exp\left[\frac{E_{ph}}{k_{B}T_{D}}\right]}{\left[\frac{V_{gap}}{V_{min}}\right]\left[\frac{V_{b}}{V_{b\_max}}\right]}}.$$

which is obtainable under the parameter set of $E_{ph},\;L$,$V_{b}$ and $T_{D}$ [41] In this latter equation, $V_{gap}=L^{3}$, $V_{min}={\left(\lambda_{ph}/2\right)}^{3}$ and $\lambda_{ph}$ the photon wavelength. In accordance with Equation (3), the maximum bias level is ${qV}_{b\_max}=2m_{e}c^{2}$. While L and $V_{b}$ determine the macroscopic observability ${OV}_{EO}$, a high level of statistical significance ${SI}_{EO}$ can independently be obtained by ensuring a sufficiently low detector operation temperature, satisfying ${k_{B}T}_{D}\ll E_{ph}$. With a detected EO fulfilling the conditions ${OV}_{EO}\gg 1$ and ${SI}_{EO}(L,V_{b},T_{D})\approx 1,$ the EO qualifies as a truly valid “*yes-no indication*” concerning the question of whether a photon had been interacting with the device or not.

**Figure 3:**
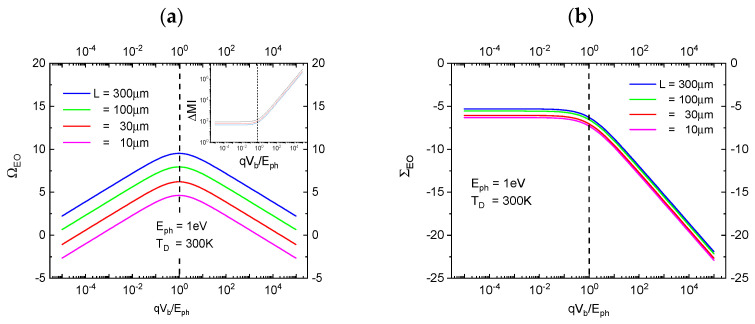
(**a**) Observability $\Omega_{EO}$ as a function of the normalized bias potential ${qV}_{b}/E_{ph}$ with the device size L as a parameter. The different curves in the inset show the impact of temperate on the entropy production; (**b**) statistical significance $\Sigma_{EO}$ as a function of the normalized bias potential ${qV}_{b}/E_{ph}$ and as evaluated for different device sizes L. For clarity of presentation the curves in Figure 3b have been slightly offset from each other.

In Figure 3a,b below, we turn to the entropic cost that is associated with the production of EOs with FOMs of ${OV}_{EO}$ and ${SI}_{EO}$, respectively. Defining entropy-normalized FOMs by [36]

(7) $$\Omega_{EO}=\frac{1}{\ln\left(2\right)}\frac{{OV}_{EO}}{{MI}_{D}},$$

and

(8) $$\Sigma_{EO}=\frac{1}{\ln\left(2\right)}\frac{{SI}_{EO}}{{MI}_{D}}$$

with their associated entropy cost being measured in terms of the missing microscopic information [38,39,40]

(9) $${MI}_{D}\left({E_{ph},V}_{b},T_{D}\right)=\frac{1}{ln(2)}\;\frac{E_{ph}+qV_{b}}{k_{B}T_{D}},$$

generated during the energy dissipation inside the photoanode in step c, Figure 3a,b below shows that both entropy-rated FOMs take on optimum values at minimum entropic cost, in case both photon and detector share evenly in the energy expense of producing a macroscopically observable EO. This latter result clearly expresses the necessity of *“observer participance”* in generating macroscopically accessible “*yes-no indications*”.

## 6. Why No Continuity Physical Laws?

So far, we have been dealing with individual EOs, the Heisenberg cut, and the energetic and entropic cost involved in the process of micro–macro amplification. As revealed by the photographs in the bottom row of Figure 1b, the final experimental answers provided by a DSE are multi-bit pieces of cognitive information that take the spatial form of “granular diffraction patterns” (GDP). Upon observing GDPs, these patterns demonstrate the second capability of photons of transporting momentum in the form of undulatory motion. This latter property is complementary to the previously demonstrated capability of photons of becoming localized in the form of apparent “particle impacts”.

With regard to the informational content and the energy expense involved in observing GDPs, the observation of the complementary property of wave-like motion is associated with a much higher energetic cost than the observation of any of those constituent single “particle impacts”. Recalling that the generation of each individual EO requires an observational energy expense of $E_{obs\_EO}\approx E_{ph}$, it is clear that the generation of a granular diffraction pattern (GDP) is associated with an energetic burden of

(10) $$E_{obs\_GDP}(N_{trial})\approx N_{trial}{\;E}_{obs\_EO},$$

where $N_{trial}$ counts the number of times a photon had been passed through the DSE to build up a GDP. Trying to push the experimental answer towards its continuum limit by increasing $N_{trial}\to \infty$, the observational energy demand is pushed towards infinity, i.e., towards an amount of energy which is practically impossible to supply. A practical example of pushing towards the limit $N_{trial}\to \infty$ and of following the trajectory of corpuscular photon is numerically illustrated in Appendix A.

At this limit of $N_{trial}\to \infty$, another problem is encountered as revealed by the series of photographs at the bottom row of Figure 1b. These photographs suggest that in the limit $N_{trial}\to \infty$, the white dots, which represent the individual EOs, will merge and ultimately produce the illusion of a continuously varying diffraction pattern that had originated from the diffraction of a classical electromagnetic wave. In this way, any evidence of the inherent quantum behavior of discontinuously and sequentially producing experimental answers from individual EOs is completely obscured. In this latter case of a practically unreachable limit of $N_{trial}\to \infty$, we again arrive at Bohr’s statement that out of each pair of complementary properties, only one is realizable by means of measurement.

To summarize, we conclude that Wheeler’s statements 1–3 about the “*impossibility of continuum idealizations of physical laws*” arises out of two reasons:

(a) The excessive growth of the observational energy demand with $N_{trial}\to \infty$, and, secondly,
(b) The loss of granularity in the experimental response that masks any indication of the underlying discontinuous quantum phenomenon.

## 7. Landauer Principle and “Observer Participance”

In the previous sections we have been looking at photons from the perspective of Bohr’s complementary principle. As seen from this point of view, photons are quantum objects which are capable of propagating in two complementary forms, either in the form of undulating electromagnetic waves or in the form of localized particles with the specific form being determined by experimental boundary conditions. In this section we take a different perspective of photons as being quantum objects which carry potential information $i_{pot}$ that can be revealed in a detection process:

(11) $$i_{pot}\left(E_{ph},T_{D}\right)=\;\frac{1}{ln(2)}\frac{E_{ph}}{k_{B}T_{D}}.$$

In the above equation, $E_{ph}$ is the photon energy, $T_{D}$ the detector temperature and $k_{B}$ Boltzmann’s constant. We have already shown in previous papers [40,41] that large values of $i_{pot}\gg 1$ lead to photon detection events with high levels of signal-to-noise ratio, or equivalently to detection events with high levels of statistical significance, i.e., events which are unlikely to have been caused by a random thermal fluctuation inside the detector itself.

In order to acquaint the reader with the concept of potential information, consider a photon with energy $E_{ph}$ moving towards the photocathode of a PID as illustrated in Figure 2a with a work function, satisfying $q\phi_{B}=E_{ph}$. With the photon energy of $E_{ph}$ being available upon absorption, an electron can be raised from the Fermi level of the photocathode to its vacuum level. As the excited electron can stay there for only a very short time, the electron will drop back onto the Fermi level, where its potential energy is rapidly converted into a large number of smaller energy packages of size ${\approx k}_{B}T_{D}$, which causes the entropy of the photocathode to increase by

(12) $$S_{anode}(E_{ph},T_{D})=\frac{E_{ph}}{T_{D}}$$

or equivalently into a piece of missing thermodynamic information [39]

(13) $$MI\left(E_{ph},T_{D}\right)=\frac{1}{ln(2)}\frac{E_{ph}}{k_{B}T_{D}}$$

concerning the internal state of motion inside the photocathode. By increasing the lack of information concerning the internal state of motion inside the photocathode, the information potential of the photon had been erased, i.e., irreversibly been annihilated. By dividing the energy $E_{ph}$ dissipated through the amount of missing information generated, the dissipative energy expense is revealed that had to be expended to erase one bit of potential information that the photon had carried with itself prior to its absorption:

(14) $$\frac{E_{ph}}{MI\left(E_{ph},T_{D}\right)}=\;\ln(2)k_{B}T_{D}=E_{La}$$

In this latter equation, $E_{La}=ln\left(2\right)k_{B}T_{D}$ is the Landauer energy [43,44,45,46,47].

In order to make a connection to the Landauer principle of information erasure [43,44,45,46,47], consider an absorption event as above; however, with the important difference that the excited electron has now been able to move during its very short lifetime at $E_{vac}$ towards the cathode surface that directly faces the photoanode. With the electron now being exposed to the externally applied electric field, the electron is rapidly drawn towards the photoanode, thereby gaining kinetic energy equivalent to

(15) $$E_{kin}=qV_{b}\approx \;E_{ph},$$

while concomitantly generating a displacement current pulse $I_{s}(t)$ which linearly increases with drift time t and which abruptly terminates as the surface of the photoanode is reached. While this displacement current pulse generates the desired macroscopically observable output signal event with an informational value of one cognitive bit, both the kinetic energy of the photoelectron of $E_{kin}\approx \;E_{ph}$ and the photon energy $E_{ph}$ itself are dissipated inside the photoanode in step c and converted into low-temperature heat there. In this way, the total entropy production inside the photoanode is raised by

(16) $$S_{cathode}≅\frac{2E_{ph}}{T_{D}}≅2S_{anode}$$

which is twice the amount of entropy that had been generated in the first scenario in which the information potential of the photon had been erased without creating any observable effect at all. Asking again for the dissipative energy expense for generating one bit of cognitive output information, it is revealed that this energy expense had now been doubled relative to the passive absorption event discussed before:

(17) $$\frac{{2E}_{ph}}{MI\left(E_{ph},T_{D}\right)}=\;{2ln(2)k}_{B}T_{D}=\;{2E}_{La}$$

In order to avoid overheating and up-charging of the photoanode, the low-temperature heat needs to be dispersed by means of heat conduction in the wider environment of the photoanode and, in addition, the thermalized photoelectron needs to be moved by means of observer-supplied energy from the Fermi energy of the photoanode up towards the Fermi energy of the photocathode to avoid up-charging of the photoanode. With erasure and reset having been implemented, the total energy expense for erasing each bit of potential information, that the photon had carried prior to its detection, has finally been increased to:

(18) $$\frac{{3E}_{ph}}{MI\left(E_{ph},T_{D}\right)}\geq {3ln(2)k}_{B}T_{D}={3E}_{La}$$

Taking an overall look at the scenarii considered above, the potential information carried by the photon prior to its absorption is irreversibly dissipated and thereby erased in all of the three cases discussed above. The expense in observer-supplied amounts of energy, however, differs in each case:

(19) $$E_{erase}=\;E_{La}$$

in case no observable event has been generated upon absorption.

(20) $$E_{erase}={2E}_{La\;}$$

in case a macroscopically observable event has been generated upon absorption and accumulated charge is not removed from the photoanode.

(21) $$E_{erase}\geq {3E}_{La}$$

in case a macroscopically observable event has been generated upon absorption and the accumulated charge has been removed from the photoanode and the PID been reset for a new round of photon detection.

While the first case (Equation (19)) refers to the case of an unobserved and unobservable re-distribution of energy inside an ideal gas or inside a macroscopic piece of solid, for instance, the latter two cases refer to situations in which an inherently unobservable quantum matter interaction has triggered an amplification process in which the unobservable micro-event had been turned into a macroscopically observable event.

Overall, the Landauer principle of information erasure reveals as another manifestation of the necessity of *“observer participance”* in turning unobservable photon–matter interactions into macroscopically observable EOs.

## 8. Summary and Conclusions

In this work we have been following Wheeler’s observational approach to physics and considered the double-slit experiment with photons (DSE) as a question posed to nature. Specifically, this experiment asks the following question: “How does a photon propagate through a DSE, i.e., a device which challenges each photon with obstacles which can be overcome with different ease, depending on whether the photon chooses to take either its wave or its particle option of propagation?” As commonly observed and widely reported in the literature, the experimental answers provided by a DSE are being built up in a sequential manner from single “particle impacts” gradually evolving into granular “diffraction patterns” (GDPs).

Following Wheeler’s mindset, we have re-interpreted the observables of “apparent particle impacts” as “elementary observations”, EOs, or quanta of observation, which represent binary pieces of cognitive information. With this association in mind, the GDPs which evolve out of EOs turn into multi-bit messages that constitute the final experimental answers. In this paper, our focus has not been on how experimental answers are impacted by “which-way” or “delayed choice” experiments. Rather, our key interest has been to reveal and to elucidate those processes which turn microscopic and principally unobservable processes of “wave function collapse” on the photographic detection screens into macroscopically observable events that enable simple binary decisions to be taken.

In order to arrive at this goal, we have mentally replaced the state-of-the art photographic detection screens with their unobservable and hard-to-understand internal photochemistry by photo-ionization detectors (PIDs), which are conceptional devices that feature easily overseeable physics, on the one hand, and a potential of performing as ideal photon detectors, on the other hand. In this way, we have been able to unravel those processes that turn principally unobservable photon–detector interactions on the length scale of quantum phenomena into macroscopically observable events.

In brief, our main findings are as follows:

- EO-initiating wave function collapses occur at the photocathode of a PID, which places the position of the so-called Heisenberg cut at the photocathode surface directly facing the photoanode.
- All other parts of conceptual PID devices are entirely devoted to supporting classical processes that turn the detection-initiating processes of “wave function collapses” into macroscopically observable output events.
- In order to support these downstream “amplification processes”, observer-supplied energy resources are required to actually enable these processes—a measure which represents an important act of “observer participance”.
- The backend “amplification processes” are associated with a measurable energy demand and an associated entropy cost, both of which are optimized in case the observer-related energy supply matches the energy of the photons that had initiated an EO.
- The operational principles of PIDs reveal that EOs are informational entities with a double nature of being both binary pieces of cognitive information, on the one hand, and physical entities measurable in units of the Planck constant of physical action h, and the Boltzmann constant $k_{B}$, which forms the base unit of entropy.
- Building on the easily overseeable physics of PIDs, a connection between Wheeler’s “Observer Participance” and the “Landauer Principle” of information erasure could be made.
- In the Landauer interpretation of detection, the formation of a microscopically observable EO is enabled by erasing in a bit-per-bit manner each thermodynamic bit of information, that an incoming photon had carried prior to its detection, at an energy demand of $E_{erase}\geq 2E_{La}$ inside the PID, with $E_{La}$ = $k_{B}T_{D}\ln\left(2\right)$ standing for the Landauer energy.

## Figures and Tables

**Figure A1:**
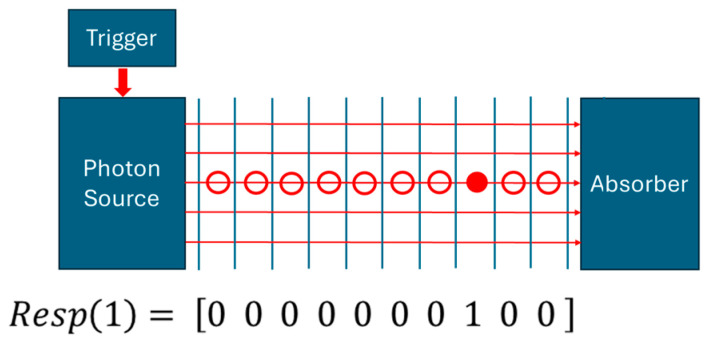
A photon source, producing plane-wave fronts carrying photons, moving on parallel paths, from source to sink. Along the chosen line, a 10-detector array is positioned at regular positions with identical distances between detectors to allow photons to be detected as they move from source to sink. In the example above, a single photon traveling through the array is randomly absorbed at the eighth detector downstream to the source, thereby producing as an output signal a 10-element response vector with ZEROs at all positions, except for position 8.

**Figure A2:**
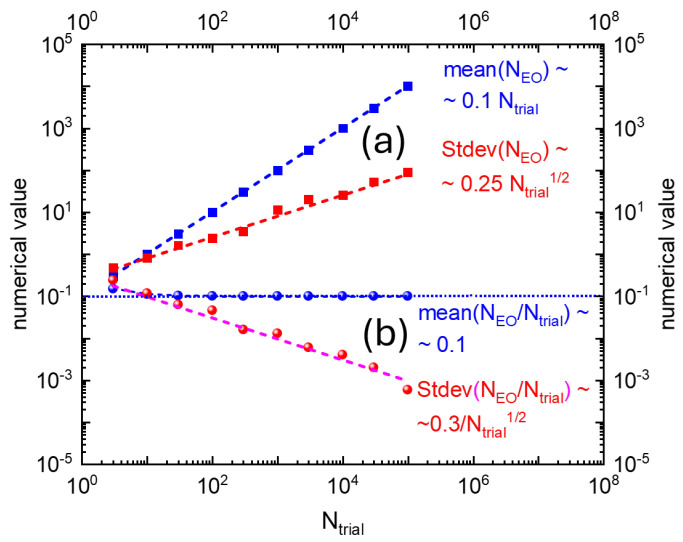
(**a**) Top curves: mean numbers of accumulated EOs (*N_EO_*) and their standard deviation (Stdev(*N_EO_*)) with increasing numbers of photon passages $N_{trial}$ through the array; (**b**) Bottom curves: same quantities as above but normalized to the numbers of photon passages $N_{trial}$ through the array.

## Data Availability

All relevant data are contained in the published text.

## Appendix A. Do Photons Travel Along Straight Lines?

After Planck [8] and Einstein [9] had proposed their photon hypothesis, the idea became popular that photons propagate through empty space along simple straight lines, similar to corpuscular particles moving through force-free environments. In this appendix we consider the example of a freely propagating photon and show how a discrete set of observations converges to the continuum limit of a simple straight line.

Figure A1 sketches a thought experiment that could be used to test the proposition made in the headline. As shown there, a plane electromagnetic wave is launched from a photon source on the left and allowed to propagate towards a photon absorber on the right. The photon source in Figure A1 can in principle be turned into a single-photon source by appropriately reducing the time-dependent electric fields used in the electromagnetic wave generation. In order to trace out the trajectories of photons emitted there, ten detectors are positioned along the imagined photon trajectory, with each detector being placed at equidistant positions Δz≫λ from its neighbor, and with $z_{i}=i\;\Delta z$ standing for the individual detector positions and λ for the photon wavelength. Providing each detector with a quantum efficiency of η=0.1, any photon emitted from the source will have been absorbed inside the array upon arriving at the downstream absorber. In order to allow following the photon trajectory, each detector inside this 10-detector array needs to be constructed in a way that a photon is able to travel freely through all detectors, with a chance of interacting with any of those detectors on its way to the absorber to produce an EO there. If absorbed at position i, the detector will produce a ONE as an output at vector position *i* and a ZERO at each vector position j≠i in case no detection occurred there. As each photon can only be absorbed once, the passage of a single photon through this array will produce a 10-element row vector with a ONE at a random position and ZEROs on all other positions, as shown in Figure A1.

Starting with the experiment, we first send three photons sequentially through the array. In this way, three 10-element row vectors are produced with ONES at random positions:

(A1) $$Resp\left(1\right)=\left[1\;0\;0\;0\;0\;0\;0\;0\;0\;0\right]\;Resp\left(2\right)=\left[0\;0\;0\;0\;0\;0\;0\;0\;1\;0\right]\;Resp\left(3\right)=\left[0\;0\;0\;0\;0\;0\;1\;0\;0\;0\right]$$

Vector addition of all three responses produces a new 10-element row vector, resulting in an average number of response indications per sensor position of ${EO}_{av}\left(3\right)$:

(A2) $$EO\left(3\right)=\left[1\;0\;0\;0\;0\;0\;1\;0\;1\;0\right];\;\;\;\;\;\;\;\;\;\;\;\;\;{EO}_{av}\left(3\right)=0.3$$

Repeating this process of passing photons and accumulating outputs, random vectors with increasingly larger natural numbers on all positions result, with relatively smaller statistical scatter between individual positions, as the number of trials $N_{trial\;}$ is increased:

(A3) $$\begin{array}{l} EO\left(10\right)=\left[0\;1\;0\;1\;2\;0\;1\;2\;2\;2\right];\;\;\;\;\;\;{EO}_{av}\left(10\right)=1.1\\ EO\left(10^{2}\right)=\left[7\;17\;6\;4\;12\;15\;9\;11\;14\;6\right];\;\;\;{EO}_{av}\left(10^{2}\right)=10.2\\ EO\left(10^{3}\right)=\left[109\;119\;83\;98\;105\;92\;85\;95\;101\;114\right];\;\;{EO}_{av}(10^{3})=110.1 \end{array}$$

Normalizing the row vectors EO with regard to the number of photons $N_{trial}$ that had been passed through the array, it is easily seen that the numbers ${EO}_{av}(N_{trial})$ converge to the quantum efficiency of each detector of η=0.1. Concomitantly, the relative scatter between vector positions decreases proportional to $N_{trial}^{-1/2}$. These dependencies as functions of $N_{trial}$ are pictorially presented in Figure A2.

Recalling that an ideal approximation to a straight line is obtained when all entries for the individual detector positions are exactly the same, Figure A2 shows that this situation is attained in the case $N_{trial}\to \infty .$ Further recalling that for generating each individual EO an observer-related energy cost of $E_{ph}$ is involved, the observational energy response raises towards infinity, i.e., a result that underlines Wheler’s statement of the “impossibility of continuum idealizations of physical laws”.
